# Simultaneous Electrochemical Determination of Chlorzoxazone and Diclofenac on an Efficient Modified Glassy Carbon Electrode by Lanthanum Oxide@ Copper(I) Sulfide Composite

**DOI:** 10.3389/fchem.2022.889590

**Published:** 2022-06-17

**Authors:** Bahar Baniahmad, Hadi Hassani Nadiki, Shohreh Jahani, Najmeh Nezamabadi-Pour, Ali Toolabi, Mohammad Mehdi Foroughi

**Affiliations:** ^1^ Department of Pharmacology and Toxicology, Isfahan Pharmaceutical Sciences Research Center, School of Pharmacy and Pharmaceutical Sciences, Isfahan University of Medical Sciences, Isfahan, Iran; ^2^ Department of Chemistry, Kerman Branch, Islamic Azad University, Kerman, Iran; ^3^ Noncommunicable Diseases Research Center, Bam University of Medical Sciences, Bam, Iran; ^4^ Bam University of Medical Sciences, Bam, Iran; ^5^ Department of Environmental Health Engineering, School of Public Health, Bam University of Medical Sciences, Bam, Iran

**Keywords:** diclofenac, chlorzoxazone, nanocomposite, voltammetry, modified electrode

## Abstract

This study synthesized a La_2_O_3_@snowflake-like Cu_2_S composite to fabricate an electrochemical sensor for sensitively simultaneous detection of diclofenac and chlorzoxazone exploiting an easy hydrothermal approach, followed by analysis with XRD, FE-SEM, and EDX methods. According to voltammetric studies, the electrocatalytic diclofenac and chlorzoxazone oxidations on the electrode modified with La_2_O_3_@SF-L Cu_2_S composites were increased, with greater oxidation currents, as well as the oxidation potential was significantly decreased due to synergetic impact of La_2_O_3_@SF-L Cu_2_S composites when compared with the pure SF-L Cu_2_S NS-modified electrode. The differential pulse voltammetry findings showed wide straight lines (0.01–900.0 μM) for La_2_O_3_ NP@SF-L Cu_2_S NS-modified electrode with a limit of detection (LOD) of 1.7 and 2.3 nM for the detection of diclofenac and chlorzoxazone, respectively. In addition, the limit of quantification was calculated to be 5.7 and 7.6 nM for diclofenac and chlorzoxazone, respectively. The diffusion coefficient was calculated to be 1.16 × 10^−5^and 8.4 × 10^−6^ cm^2^/s for diclofenac and chlorzoxazone oxidation on the modified electrode, respectively. Our proposed electrode was examined for applicability by detecting diclofenac and chlorzoxazone in real specimens.

## 1 Introduction

The treatment of osteoarthritis and rheumatoid arthritis as well as the relief of painful symptoms associated with musculoskeletal conditions are now possible with the simultaneous administration of many drugs. Thus, single-pill combinations (SPCs) were formulated to facilitate administration. This issue emphasizes the development of effective analytical approaches for the simultaneous detection of coadministered drugs in the presence of associated agents and impurities. One of the SPCs is a combination of diclofenac sodium and chlorzoxazone (Ahmed et al., 2020).

Chlorzoxazone (5-chloro-3H-benzooxazol-2-one) as a potent muscle relaxant is prescribed for painful musculoskeletal conditions with central function. The drug primarily affects the spinal cord and subcortical areas, thus inhibiting multisynaptic reflex arcs implicated in the production and maintenance of skeletal muscle spasms of various causes. The reduction of muscle tone and tension, and thus relief of spasms and pain associated with musculoskeletal disorders can occur with this drug (Abbar and Nandibewoor, 2012a). Sodium [o- (2,6-dichloroanilino) phenyl] acetate (diclofenac sodium or DS) belongs to the nonsteroidal anti-inflammatory drug (NSAID), which exhibits anti-inflammatory, analgesic, and antipyretic activity (Eteya et al., 2019). It is prescribed to manage many painful and inflammatory diseases, including renal colic, rheumatoid arthritis, soft tissue disorders, osteoarthritis, acute gout, migraine, and dysmenorrhea (Sweetman, 2005; Al-Turki et al., 2010). Concomitant administration of diclofenac sodium and chlorzoxazone is used to relieve moderate to severe pain associated with musculoskeletal spasms. Despite all this, serious side effects have been reported for this formulation such as heart attack and stroke, especially in long-term administration. Other reported side effects include gastrointestinal disorders, aplastic anemia, renal dysfunction, and agranulocytosis (Sweetman, 2005). Accordingly, the detection of trace amounts of these drugs in biological and pharmaceutical media is essential for therapeutic purposes and drug production. Various techniques were employed for the simultaneous detection of diclofenac and chlorzoxazone, such as absorbance difference spectrophotometry (Saheb et al., 2004), the spectrophotometric Q-absorbance ratio method (Patel and Prajapati, 2013), and the HPLC technique (Ahmed et al., 2020) exploiting CN-bonded phase column. However, the disadvantages of these methods are the need for derivatization and the time required for extraction (Zhang et al., 2010; Jahani, 2018; Setoudeh et al., 2020).

Among these, voltammetry is the proposed method for bypassing such problems due to its unique advantages such as rapid response, inherent selectivity, great sensitivity, simplicity, cost-effectiveness, and relatively short analysis time to determine organic molecules such as drugs and relevant molecules in biological fluids and pharmaceutical doses (Maaref et al., 2018; József et al., 2019; Salajegheh et al., 2019; Sargazi et al., 2019; Shetti et al., 2019; Kulkarni et al., 2020; Malode et al., 2020; Vakili Fathabadi et al., 2020; Liu et al., 2021a; Foroughi et al., 2021; Obireddy and Lai, 2021; Obireddy and Lai, 2022). An effective strategy to increase the electrochemical performance of sensors is to design composites by integrating highly electrocatalytic active materials with excellent conductivity materials, resulting in the electrode surface modification (Zhang et al., 2016; Foroughi and Ranjbar, 2017; Shetti et al., 2018; Fathi et al., 2020; Azizabadi et al., 2021; Liu et al., 2021b; Li et al., 2021; Liang et al., 2022; Qi et al., 2022).

Much research is currently being done on semiconductor nanostructures as part of active and integrated electrochemical nano-devices. Selecting a semiconductor material possessing a suitable bandgap is the most basic action in this research. In addition, if a material is abundant, non-toxic, green, and recyclable, it can be easily grown into various nanostructures using cost-effective techniques on different substrates (Mousavi-Kamazani et al., 2013; Guan et al., 2020). One of the suitable options to meet the aforementioned conditions with a 1.21-eV bandgap is copper (I) sulfide. Depending on the stoichiometric properties, copper sulfides can take many crystalline phases, from copper-rich Cu_2_S phases to copper-poor CuS_2_ phases (Mousavi-Kamazani et al., 2016). The pure production of each phase is of particular importance, especially in the case of chalcocite copper (I) sulfide (Cu_2_S) which is a widely used material in various fields (Kristl et al., 2013; Brewer and Arnold, 2014).

Lanthanum oxide (La_2_O_3_) is one of the important oxides belonging to the family of rare earth oxides. This p-type semiconductor with a 5.5-eV bandgap has been previously applied in different fields, such as in luminescence equipment, electromagnetic devices, super-capacitors, photoelectro-chemical cells, catalysts, gas sensors, and Li-ion batteries (Larsen et al., 2003). In addition to these advantages, La_2_O_3_ is a promising candidate as an alternative to the production of green sensors in detecting a variety of pollutants due to its unique properties such as greater capacity, admirable chemical and thermal stability, large dielectric constant (ε) of 27, less toxicity, broad voltage range, and less oxide leakage current. A significant elevation has been reported in the catalytic activity mediated by nanocomposites containing La_2_O_3_ loaded on various nanoparticles (NPs) (Lee et al., 2015; Zhou et al., 2016; Li et al., 2017; Yadav et al., 2017; Salinas et al., 2018; Jaffar et al., 2019; Umar et al., 2020).

For the first time, we synthesized and characterized the effective material, La_2_O_3_ nanoparticles (NPs)@snowflake(SF)-like Cu_2_S nanostructure (La_2_O_3_ NP@SF-L Cu_2_S NS composite), to modify the glassy carbon electrode. In addition, the literature review showed no studies on the electroanalysis and simultaneous detection of diclofenac and chlorzoxazone exploiting modified electrodes with novel nanocomposites. Voltammetric peaks were determined for diclofenac and chlorzoxazone on the proposed modified electrode. Great sensitivity and low limit of detections (LOD) were reported for these species because of the potent electrocatalytic potentials of La_2_O_3_ NP@SF-L Cu_2_S NS. The analytical behaviors of this sensor were examined for the simultaneous detection of diclofenac and chlorzoxazone using the voltammetric method. At last, the applicability of this sensor was determined to detect these compounds in the real samples.

## 2 Experimental

### 2.1 Solutions and Reagents

Sigma-Aldrich (Sigma-Aldrich, United States) was the selected company to prepare diclofenac, chlorzoxazone, ethylenediamine, thiourea ((NH_2_)_2_CS), lanthanum chloride hexahydrate (LaCl_3_·6H_2_O), and copper(II) chloride dihydrate (CuCl_2_·2H_2_O). Diclofenac and chlorzoxazone solutions (1.0 × 10^−2^ M) were applied freshly by pouring certain levels of diclofenac and chlorzoxazone into distilled deionized water (100 ml) in a volumetric flask, which was maintained in dark at cool condition. The serial dilution was performed with phosphate buffer solution (PBS) to prepare more dilute solutions. The stock solutions of 0.1 M Na_2_HPO_4_ and 0.1 M NaH_2_PO_4_ were blended to prepare 0.1 M PBS and then adjusted to the desired pH *via* NaOH or HCl. All used reagents had analytical grade, and double distilled deionized water was utilized to prepare the solutions. No more purification was performed for any of the chemicals.

### 2.2 Equipment

X-ray diffractometer (Philips analytical PC-APD), radiation of graphite mono-chromatic Cu (α_1_, *λ*_1_ = 1.54056 Å), and radiation of Kα (α_2_, *λ*_2_ = 1.54439 Å) were used for X-ray powder diffraction (XRD) performed for the structural analysis of the product. The La_2_O_3_@SF-L Cu_2_S composite morphology was analyzed by using a KYKY-EM3200 digital scanning electron microscope (SEM). The chemical composition was determined by EDX spectrometry. SAMA500 electroanalyzer (SAMA Research Center, Iran) equipped with a personal computer was applied to carry out the electrochemical measurements. The used three-electrode system included an unmodified or modified glassy carbon electrode (GCE) as the working electrode, a saturated calomel electrode (SCE) as the reference electrode, and Pt wire as the auxiliary electrode. All electrochemical measurements were under a pure nitrogen atmosphere at an ambient temperature.

### 2.3 Fabrication of the Proposed La_2_O_3_@SF-L Cu_2_S Composites

In the present study, a simple hydrothermal protocol was performed to construct La_2_O_3_@SF-L Cu_2_S composites. Thus, LaCl_3_·6H_2_O (0, 10, 20, and 30 wt%) and 170.48 mg of CuCl_2_·2H_2_O were poured into 50 ml of ethylenediamine. For this, 228.36 mg of (NH_2_)_2_CS was appended to obtain well-dispersed reactants while constantly stirring for 2 h, followed by placing a Teflon-lined stainless steel autoclave (150 ml) at different temperatures of 70, 80, and 90°C for 8 h. Next, the solutions were cooled down and the resulting products were removed and washed several times with distilled water and ethanol, followed by drying in an oven at 80°C for 8 h and storing for further testing.

### 2.4 Fabrication of Modified Electrode

The pretreatment of the GCE was performed using 0.05 μm of alumina slurry on a polishing cloth, and then washed with water and sonicated for 5 min in water. The matters adsorbed on the electrode surface were removed through ultrasonication in ethanol and double-distilled water for 5 min. The bare GCE (BGCE) modification was carried out by La_2_O_3_@SF-L Cu_2_S composites. Then, La_2_O_3_@SF-L Cu_2_S composites (1 mg) were distributed and ultrasonicated for an hour for the collection of stock solution with La_2_O_3_@SF-L Cu_2_S composites in 1 ml of an aqueous solution. The concentration of suspension is 1 mg/1 ml. Next, an aliquot of La_2_O_3_@SF-L Cu_2_S composites/H_2_O suspension solution (5 µl) was placed on the carbon working electrode, and subsequently, the solvent was evaporated at the ambient temperature.

### 2.5 Electrochemical Approach (Characterization and Testing)

Cyclic voltammetry (CV), chronoamperometry (CHA), and differential pulse voltammetry (DPV) were applied for electrochemical studies and quantification of diclofenac and chlorzoxazone, respectively.

CV is performed to a phosphate buffer (0.1 M, pH = 7.0) with and without the presence of diclofenac and chlorzoxazone (275.0 μM), starting at the equilibrium potential in the anodic direction using a potential window of 0.24–0.93 V at different scan rates. Anodic peaks are analyzed in order to establish the relation between the maximum current intensity of the anodic peaks with the scan rate.

Under optimized conditions, CHA experiments were carried out at an applied potential of 0.63 and 0.89 V *versus* SCE using different concentrations of diclofenac and chlorzoxazone, respectively.

In order to achieve the higher analytical response (anodic current), the optimal conditions for DPV measurements were as follows: PBS, pH 7.0, modulation amplitude of 0.02505 V, modulation time of 30 ms, interval time of 200 ms, step potential of 10 mV, initial potential = 200 mV, and end potential of 940 mV. To achieve the DP voltammograms of diclofenac and chlorzoxazone, appropriate volumes of the stock solutions of drugs were added to the cell containing supporting electrolytes on total bulk of 25 ml.

### 2.6 Analysis of Real Specimens

We procured the tablet solutions by completely powdering and combining five diclofenac (labeled 100 mg, Shafa Co., Tehran: Iran). Then, we used the ultrasonic bath for 4 min in order to dissolve a sufficient volume of the resulting fine powders of diclofenac in 0.1 M phosphate buffer pH = 7.0. Consequently, various volumes of all solutions have been transferred into a 25.0 ml voltammetric cell, and a standard addition procedure has been used to analyze diclofenac.

The human samples (urine and blood serum) were collected from healthy subjects and stored at −20°C for next testing. In the experiments, 2.5 ml of samples were poured into PB (22.5 ml)-containing vials, and then added with a certain volume of diclofenac or chlorzoxazone stock solution and subsequently poured into electrochemical cells.

## 3 Results and Discussion

### 3.1 Characterization of La_2_O_3_ NP@SF-L Cu_2_S NS Composite

**FIGURE 1:**
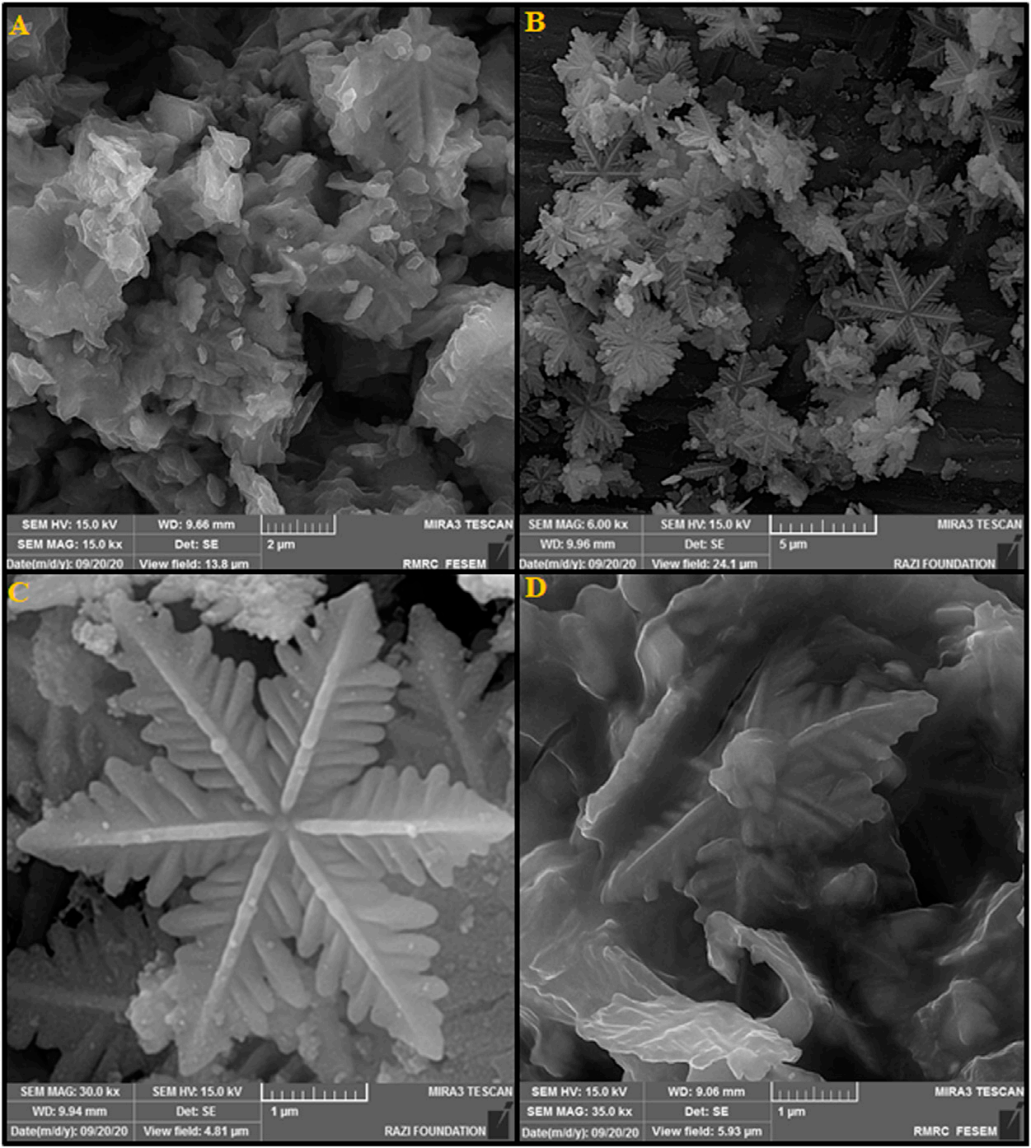
FESEM images of the La_2_O_3_ NP@SF-L Cu_2_S NS composite under various reaction temperatures: **(A)** 70°C, **(B,C)** 80°C, and **(D)** 90°C.

The SF-L Cu_2_S NS formation was confirmed by the SEM images. It was found that Cu_2_S is composed of one or more dendrites at 70°C, as shown in Figure 1A. Figures 1B,C (SEM images) show snowflake structures for the as-produced Cu_2_S specimens at 80°C. Images of a snowflake with high magnification (Figure 1C) reveal a pattern with 6x symmetry so that different large ferns emerge from a common center, which is branched in the center and along an angle of 60° from the center. The six dendritic petals have a length of approximately 2–3 µm. Further details obviously show a long central backbone and highly symmetrical secondary branches for each fern that grows preferably in two distinct directions rather than randomly. Figure 1C shows unprecedented six-branched dendrites and more branch growth. SF-L Cu_2_S NS is broken down with increasing reaction temperature up to 90°C and forms a sheet-like morphology (Figure 1D). Different reaction temperatures create significant differences in the morphology of Cu_2_S owing to the degree of effective coordination of ethylenediamine. The [Cu(En)_2_]^2+^ complex is formed by ethylenediamine *via* the copper cation at 80°C, which may initially form the nucleus and crystalline growth of Cu_2_S, and subsequently the snowflake structures. Because of the volatility of ethylenediamine, the solution forms the reduced complex at higher reaction temperatures up to 90°C. Therefore, the morphology of the as-produced Cu_2_S specimens is influenced by higher temperatures.

**FIGURE 2:**
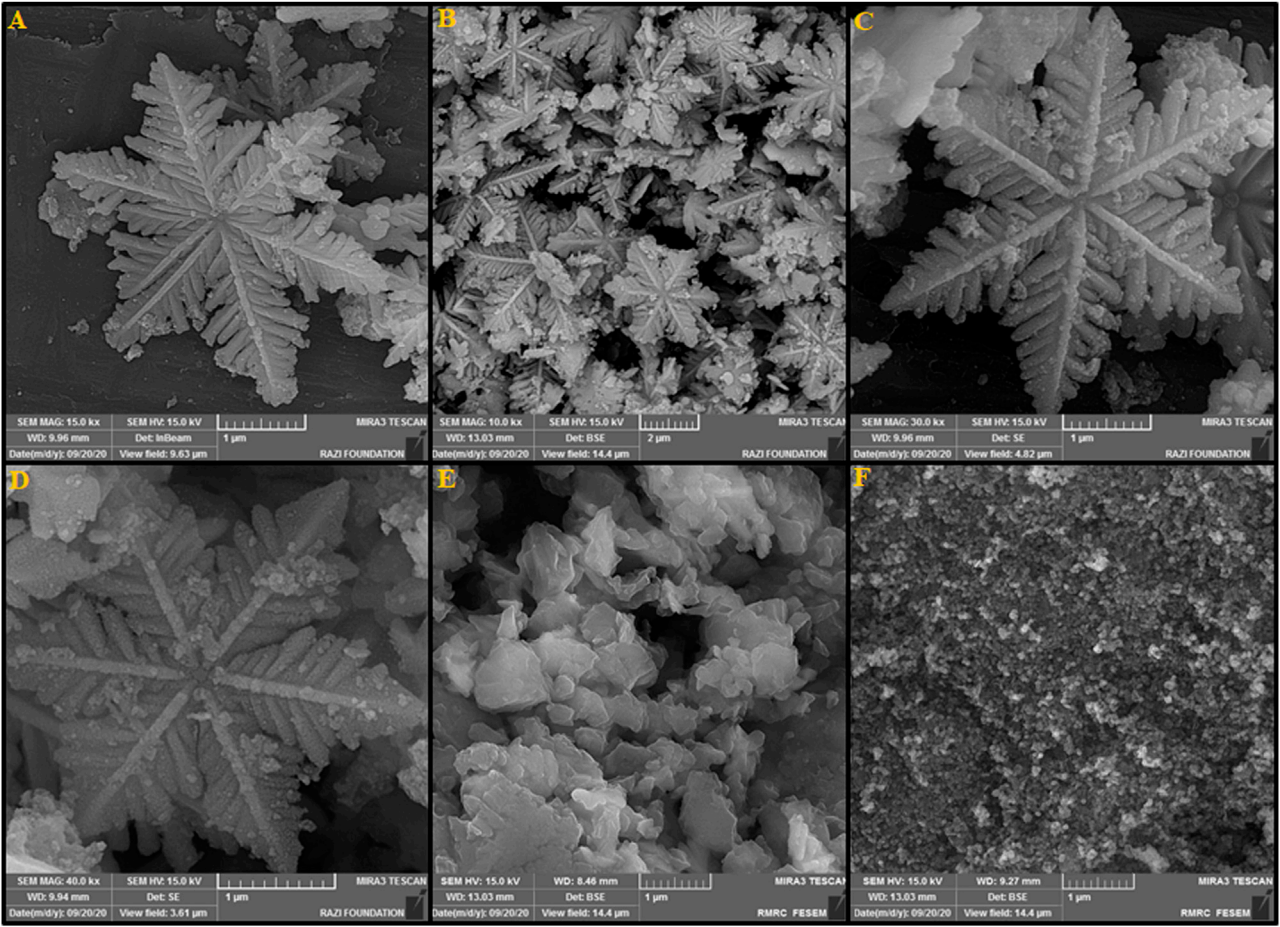
FESEM images of the La_2_O_3_ NP@SF-L Cu_2_S NS composite with different La_2_O_3_ loading amounts: **(A)** 10 wt% La_2_O_3_, **(B–D)** 20 wt% La_2_O_3_, and **(E)** 30 wt% La_2_O_3_. **(F)** The FESEM image of La_2_O_3_ NP.

The SEM images taken from La_2_O_3_@SF-L Cu_2_S composites in the presence of different loading levels of La_2_O_3_ are shown in Figure 2. Limited NPs have been gathered onto the SF-L Cu_2_S NS surface, for La_2_O_3_@SF-L Cu_2_S composites with 10 wt% La_2_O_3_ (Figure 2A). According to Figures 2B–D showing the SEM images taken from La_2_O_3_@SF-L Cu_2_S composites with 20 wt% La_2_O_3_, the SEM images display the limited-layered La_2_O_3_ NP coating on the SF-L Cu_2_S NS surface in the case of right amount of La_2_O_3_ loading, which means ultrathin La_2_O_3_ NPs similar to pure La_2_O_3_ NP as seen in Figure 2F. For La_2_O_3_@SF-L Cu_2_S composites (30 wt% La_2_O_3_), the amount of La_2_O_3_ precursors is critical as probably because of aggregating to form NPs (Figure 2E), the snowflake-like structure is completely destroyed and forms an amorphous mass that suppresses the catalytic activity of the catalysts. Hence, the optimal wt% was selected to be 20 wt% La_2_O_3_ for next electrochemical testing.

**FIGURE 3:**
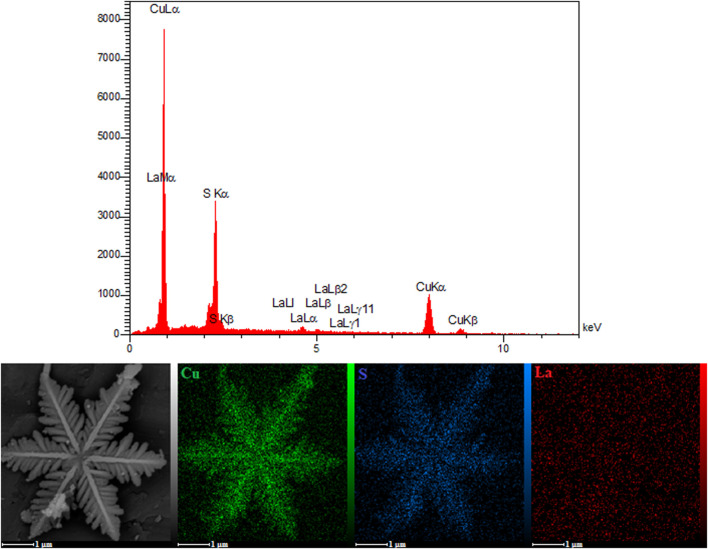
EDX spectra and elemental mapping of the La_2_O_3_ NP@SF-L Cu_2_S NS composite.

As seen in Figure 3, the EDS analysis and the mapping images for the La_2_O_3_ NP@SF-L Cu_2_S NS composite with 20 wt% La_2_O_3_ confirmed uniform dispersion of each Cu, S, and La elements, and also uniform aggregation of the La_2_O_3_ NPs on the SF-L Cu_2_S NS surface.

**FIGURE 4:**
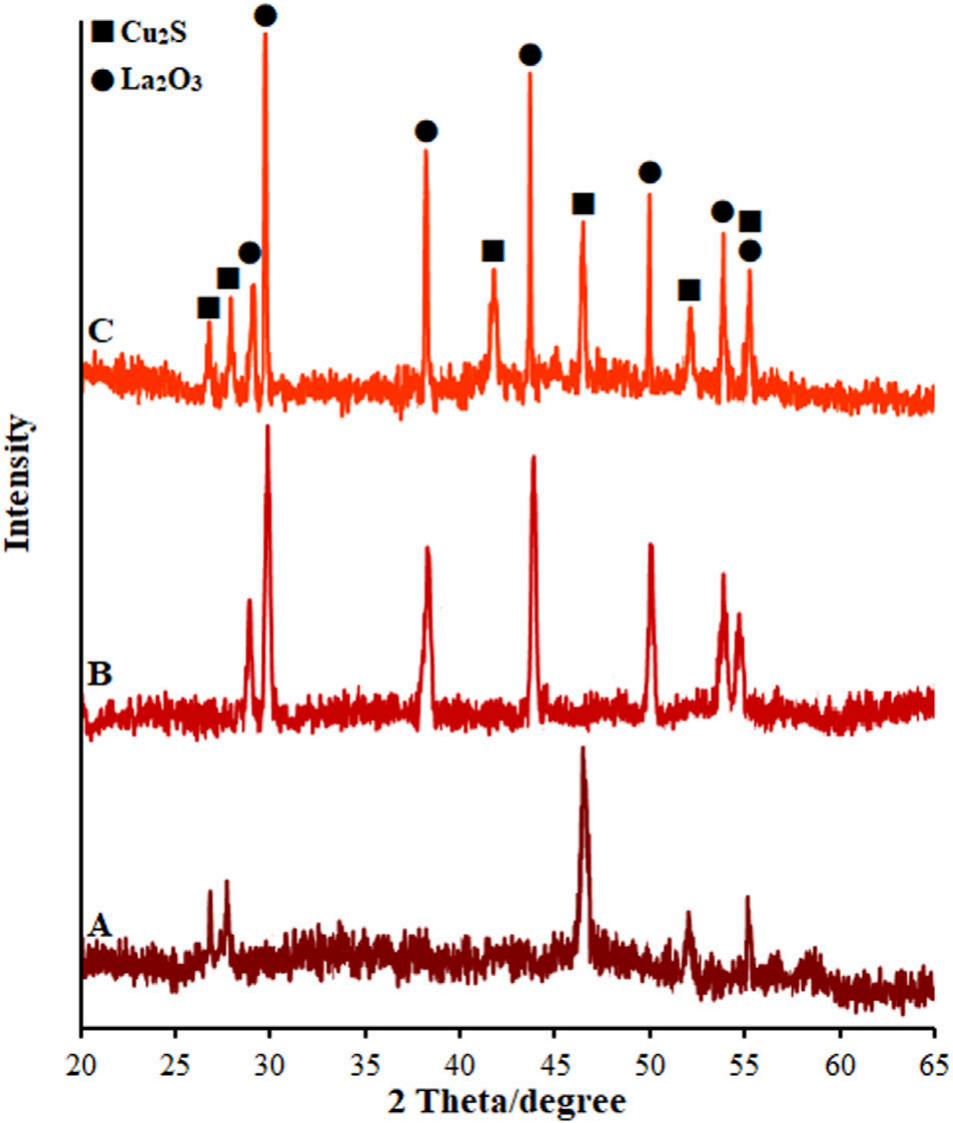
XRD pattern of **(A)** SF-L Cu_2_S NS, **(B)** La_2_O_3_ NP, and **(C)** La_2_O_3_ NP@SF-L Cu_2_S NS composite (20 wt% La_2_O_3_).

Figure 4A shows the XRD spectra taken from the as-produced SF-L Cu_2_S NS at 80°C. The positions of the XRD peaks prepared from the specimens are well consistent with the theoretical amounts of the orthorhombic stage of Cu_2_S (JCPDS 02–1294) (Chen et al., 2008). There were not any characteristic peaks as a result of any impurity, highlighting the excellent product purity. The intensity and sharpness of the peaks of XRD show well-crystallized Cu_2_S, and therefore decreased resistance of electron transfer. The XRD spectra from La_2_O_3_ NPs (La_2_O_3_ NP) are shown in Figure 4B. Based on the XRD patterns, various diffraction peaks can be seen for La_2_O_3_ NP with the polycrystalline morphology. Thus, each La_2_O_3_ NP diffraction peak is fully indexed in the structure of the quadrilateral crystal system (JCPDS Case No. 83–1348), highlighting a pure phase for the powder with hexagonal La_2_O_3_. Figure 4C attributes all sharp and obvious peaks at La_2_O_3_ NP@SF-L Cu_2_S NS patterns containing various amounts of La_2_O_3_ NP loading with 20 wt% La_2_O_3_ NP to orthorhombic Cu_2_S (JCPDS 02–1294) and hexagonal La_2_O_3_ (JCPDS 83–1348) (Kabir et al., 2018), which means successful fabrication of La_2_O_3_@SF-L Cu_2_S composites.

### 3.2 Electrochemical Characterization of the La_2_O_3_@SF-L Cu_2_S/GCE Sensor

**FIGURE 5:**
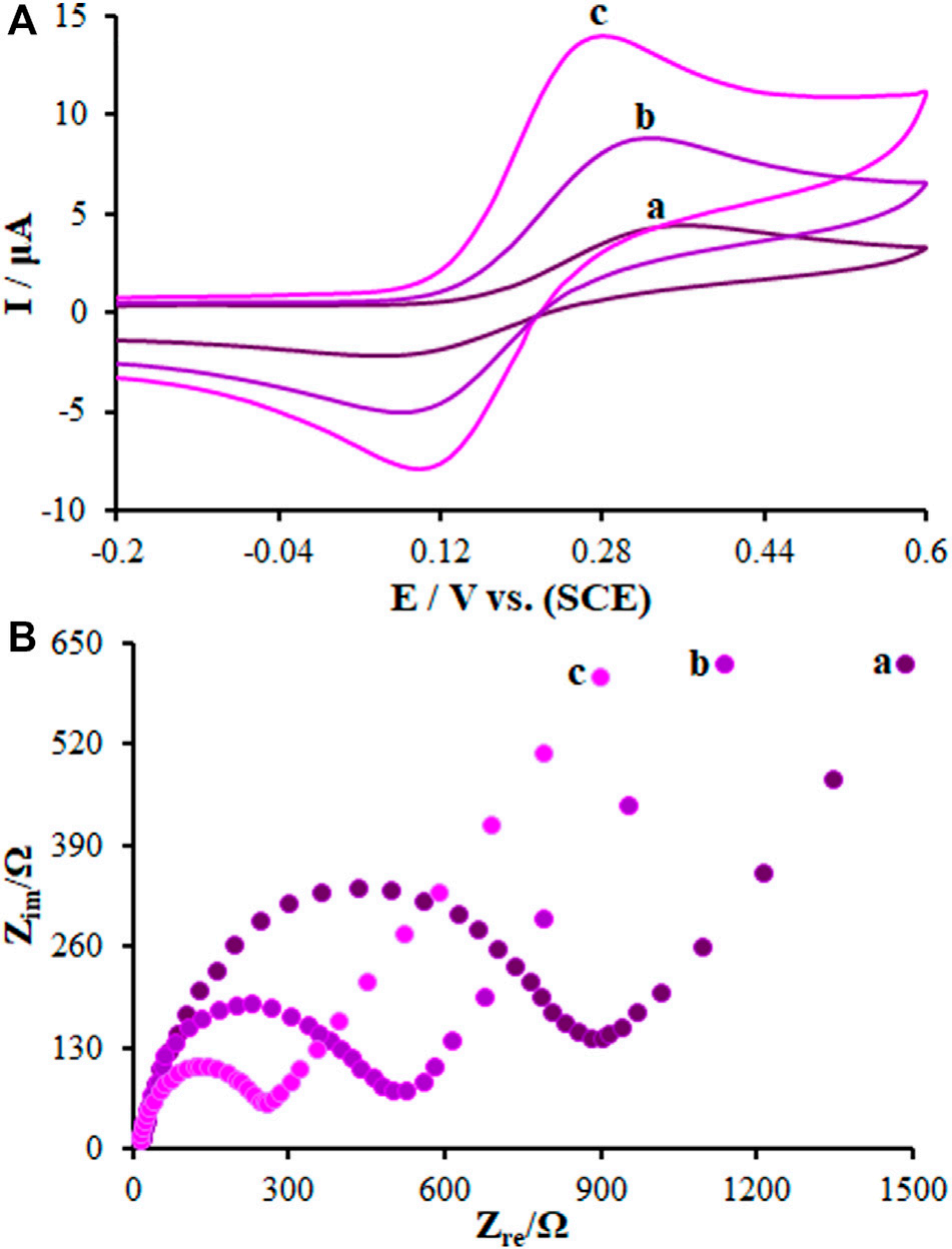
**(A)** CVs of (a) BGCE, (b) SF-L Cu_2_S/GCE, and (c) La_2_O_3_@SF-L Cu_2_S/GCE in the presence of 0.4 mM [Fe(CN)6]^3-^ solution in aqueous 0.1 M KCl. **(B)** EIS diagrams for 0.1 mM [Fe(CN)6]^3-^ solution at (a) BGCE, (b) SF-L Cu_2_S/GCE, and (c) La_2_O_3_@SF-L Cu_2_S/GCE in aqueous 0.1 M KCl. Frequency range 100 KHz to 0.1 Hz.

The CV curves for a bare GCE, SF-L Cu_2_S/GCE, and La_2_O_3_@SF-L Cu_2_S/GCE in the redox probe were plotted for the characterization of the modified GCE surface. As seen in Figure 5A, two reversible redox peaks are observed for a bare GCE, SF-L Cu_2_S/GCE, and La_2_O_3_@SF-L Cu_2_S/GCE, with a peak-to-peak difference (ΔEp) of 0.29, 0.24, and 0.17 V, respectively. The La_2_O_3_@SF-L Cu_2_S/GCE had an enhancement in the peak current corresponding to greater porosity of the La_2_O_3_@SF-L Cu_2_S composite-modified electrode surface. The redox probe electron transfer on the modified electrode surface can be facilitated by such excellent morphology of La_2_O_3_@SF-L Cu_2_S composites. The properties of La_2_O_3_@SF-L Cu_2_S composites were electrochemically determined using the EIS approach. In this method, the charge–transfer resistance (Rct) is a factor to monitor the redox probe electron transfer kinetics at the electrode interface, which means substrate attachment on the modified electrode surface. Figure 5B shows Nyquist plots of the bare GCE, SF-L Cu_2_S/GCE, and La_2_O_3_@SF-L Cu_2_S/GCE in the redox probe. Figure 4B shows a large semicircular part for bare GCE at high frequencies, as a great charge transfer resistance (Rct = 1248 Ω) corresponding to low charge and mass transfer rate on the bare GCE surface. Dramatically reduced Rcts (561 and 329 Ω) were calculated for SF-L Cu_2_S NS and La_2_O_3_@SF-L Cu_2_S composites loaded on the GCE surface, probably due to the potent ability of SF-L Cu_2_S NS and La_2_O_3_@SF-L Cu_2_S composites to amplify the electron transfer and enhance the electrode surface area.

**FIGURE 6:**
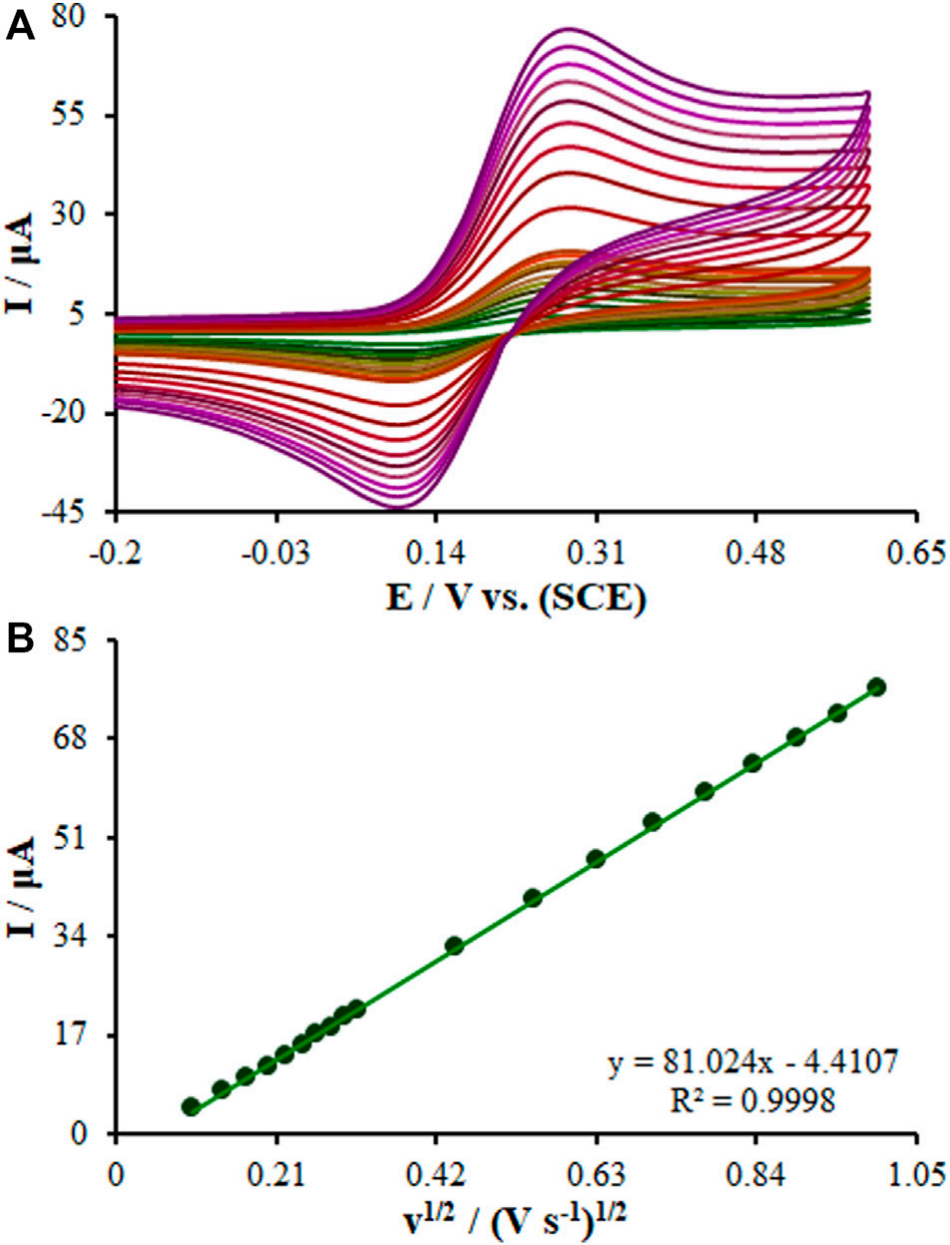
**(A)** CVs of La_2_O_3_@SF-L Cu_2_S/GCE in the presence of 0.4 mM [Fe(CN)6]^3-^ solution in aqueous 0.1 M KCl at various scan rates (from inner to outer curve): 10, 20, 30, 40, 50, 60, 70, 80, 90, 100, 200, 300, 400, 500, 600, 700, 800, 900, and 1000 mV/s. **(B)** The plot of peak currents vs υ1/2.

The Randles–Sevcik Eq. 1 was computed to analyze the efficacy of the embedded sensor, the bare GCE, SF-L Cu_2_S/GCE, and La_2_O_3_@SF-L Cu_2_S/GCE (Figure 6) (Bard and Faulkner, 2001):

$$I_{p}=\pm \left(2.69\times 10^{5}\right)n^{3/2}AD^{1/2}Cv^{1/2},$$ (1)

where all symbols possess normal meaning. The A values are 0.082, 0.17, and 0.27 cm^2^ for the surfaces of the bare GCE, SF-L Cu_2_S/GCE, and La_2_O_3_@SF-L Cu_2_S/GCE, respectively.

The electrode surface modification was evaluated by calculating the standard heterogeneous rate constant (*k* ^*0*^) based on the EIS, Eq. 2 (Bard and Faulkner, 2001):

$$k^{0}=\frac{RT}{F^{2}R_{ct}AC},$$ (2)

where *k* ^*0*^ stands for the rate constant for electron standard transfer that is heterogeneous (cm/s), *R* for global gas constant (squared with 8.314 J/K/mol), *T* for the temperature of the thermodynamic process (298.15 K), *F* for the Faraday constant values (96.485 C/mol), *R*_*ct*_ for electron transfer resistance (Ω), *A* for the electrode surface area (cm^2^), and *C* for the concentration of 0.1 mM [Fe(CN)_6_]^3-/4-^ solution.

The *k* ^*0*^ values were 2.6 × 10^−2^, 2.8 × 10^−2^, and 3.0 × 10^−2^ cm/s for the bare GCE, SF-L Cu_2_S/GCE, and La_2_O_3_@SF-L Cu_2_S/GCE, respectively. K^0^ values approach the redox couple’s kinetic potential. Therefore, a system with a higher value of k^0^ balances under lower time conditions compared to that with a lower value of k^0^, so this will be a longer balance. Hence, a higher value of k^0^ would be achieved than La_2_O_3_@SF-L Cu_2_S/GCE > SF-L Cu_2_S/GCE > GCE as for the La_2_O_3_@SF-L Cu_2_S/GCE sensor, which means greater swift electron transfer than the other electrodes.

### 3.3 Voltammetric Responses of Diclofenac and Chlorzoxazone

**FIGURE 7:**
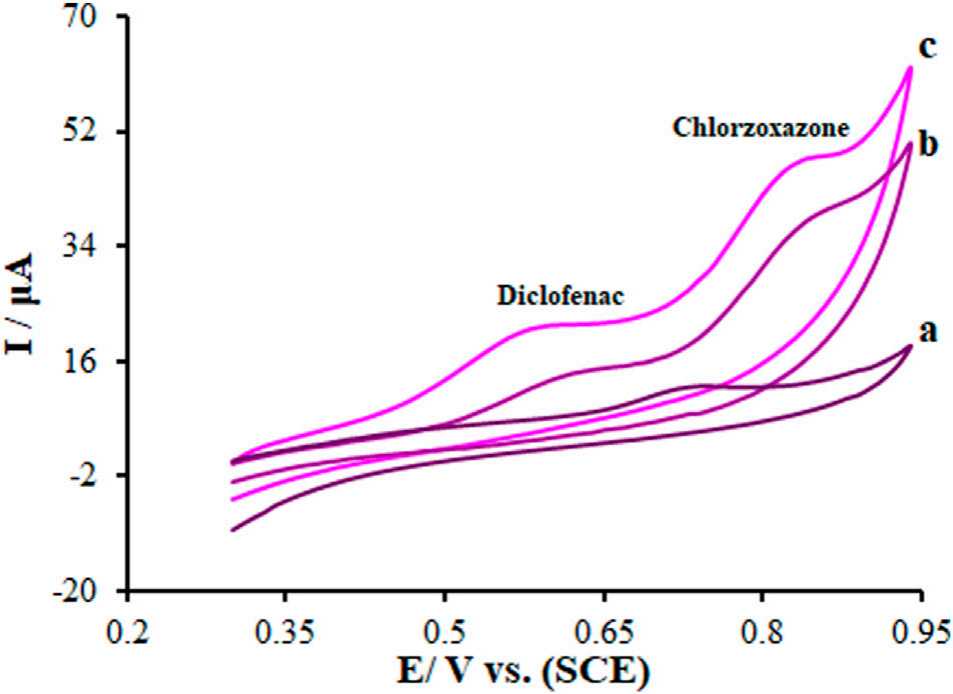
CVs of **(A)** BGCE, **(B)** SF-L Cu_2_S/GCE, and **(C)** La_2_O_3_@SF-L Cu_2_S/GCE in the presence of diclofenac (275.0 µM) and chlorzoxazone (275.0 µM) at a pH 7.0, respectively. In all cases, the scan rate was 50 mV s^−1^.

Electrochemical responses of diclofenac and chlorzoxazone (275.0 μM each) mixture in 0.1 M PBS (pH = 7) on the La_2_O_3_@SF-L Cu_2_S/GCE, SF-L Cu_2_S/GCE, and bare GCE (BGCE) surfaces were analyzed using the cyclic voltammetry (CV) method. Based on Figure 7 (curve a), the diclofenac and chlorzoxazone oxidation peaks merged (0.73 V) with a very low peak current at the BGCE. On the other hand, two clear peaks were seen for diclofenac and chlorzoxazone on the modified SF-L Cu_2_S/GCE at 0.64 and 0.86 V, respectively (Figure 7 (curve b)). According to Figure 7 (curve b), the peak currents for diclofenac and chlorzoxazone on SF-L Cu_2_S/GCE are many times higher than BGCE due to the catalytic activity of SF-L Cu_2_S NS. In addition, in Figure 7 (curve c), the potential of the peaks was shifted to a less positive potential (0.59 and 0.84 V for diclofenac and chlorzoxazone, respectively) than SF-L Cu_2_S/GCE and BGCE. Figure 7 (curve c) shows an elevation in the peak current following the augmentation of La_2_O_3_ NP to the SF-L Cu_2_S NS due to the large surface area and high conductivity of La_2_O_3_@SF-L Cu_2_S composites. The separation rates of diclofenac–chlorzoxazone oxidation peak potentials were estimated at 0.25 V, which were sufficient for the simultaneous detection of these compounds.

### 3.4 The pH Effect on Diclofenac and Chlorzoxazone Oxidation

**FIGURE 8:**
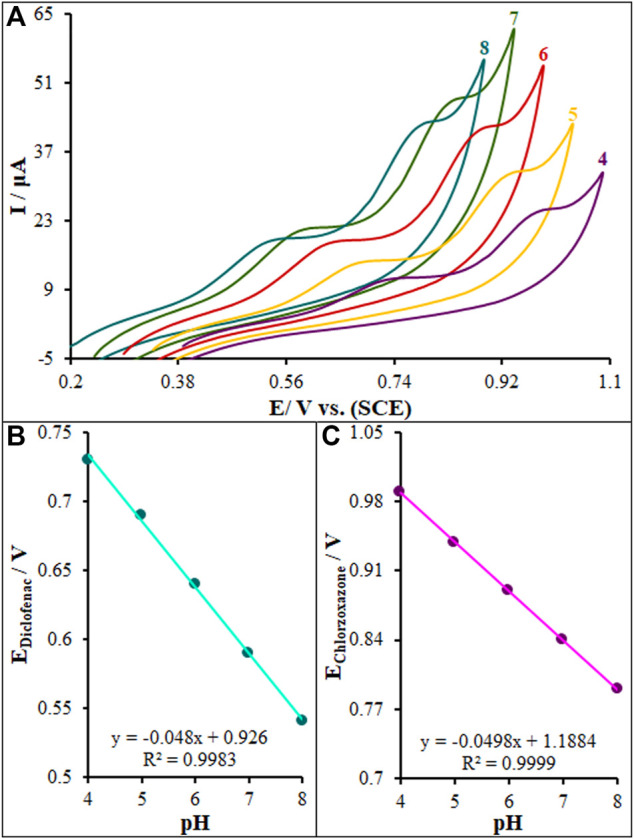
Effect of pH on the peak current for the oxidation of diclofenac (275.0 µM) and chlorzoxazone (275.0 µM); pH = 4–8. In all cases, the scan rate was 50 mV s^−1^.

The electrolyte pH significantly affects the electrooxidation of diclofenac and chlorzoxazone due to the participation of protons in the electrode reaction. The CV method was performed to analyze the pH impact on the signal of La_2_O_3_@SF-L Cu_2_S/GCE exploiting buffer solutions (0.1 M) at different pH values of 4.0–8.0, and the results can be seen in Figure 8. A slight elevation occurred in the peak currents of diclofenac and chlorzoxazone by elevating the solution pH until 7.0 and then reduction. The pH value of 7.0 resulted in the greatest peak current for the two compounds. A gradual increase in the solution pH caused the shift of peak oxidation potential of diclofenac and chlorzoxazone to less positive values, indicating the participation of protons in the electrode reactions. The PBS at the pH value of 7.0 caused the optimal reaction for peak current and peak shape, and negatively shift, suggesting pH 7.0 as the best value for subsequent tests. Figure 8B shows the plot of E_p_ *versus* pH for diclofenac and chlorzoxazone in the working pH range. There was a linear relationship between E_p_ values of two compounds and buffer solution pH, as follows:

$$\begin{array}{ccc} \text{Diclofenac:} & {\text{E}}_{\text{p}}\left(V\right)=-0.048pH+0.926 & \left(R^{2}=0.9983\right)\\ \text{Chlorzoxazone:} & {\text{E}}_{\text{p}}\left(V\right)=-0.0498pH+1.1884 & \left(R^{2}=0.9999\right) \end{array}$$

As for slopes of 0.048 and 0.0498 mV/pH for diclofenac and chlorzoxazone, respectively, they were close to the predicted Nernstian value for an equal electron and proton electrochemical process (Bard and Faulkner, 2001). In conclusion, equal numbers of protons and electrons play a role in the electrode processes.

In the proposed method, the total involvement of protons and electrons of diclofenac was found to be two. So, the following Scheme S1 was predicted as a likely electrooxidation mechanism of diclofenac. In addition, chlorzoxazone oxidation is a one-electron one-proton process, the possible product of oxidation is found to be 2-amino-4-chloro-phenol with the liberation of carbon dioxide, and the mechanism is as shown in Scheme S2. These observations were in accordance with earlier reports (Abbar and Nandibewoor, 2012a; Honakeri et al., 2020).

**SCHEME 1:**
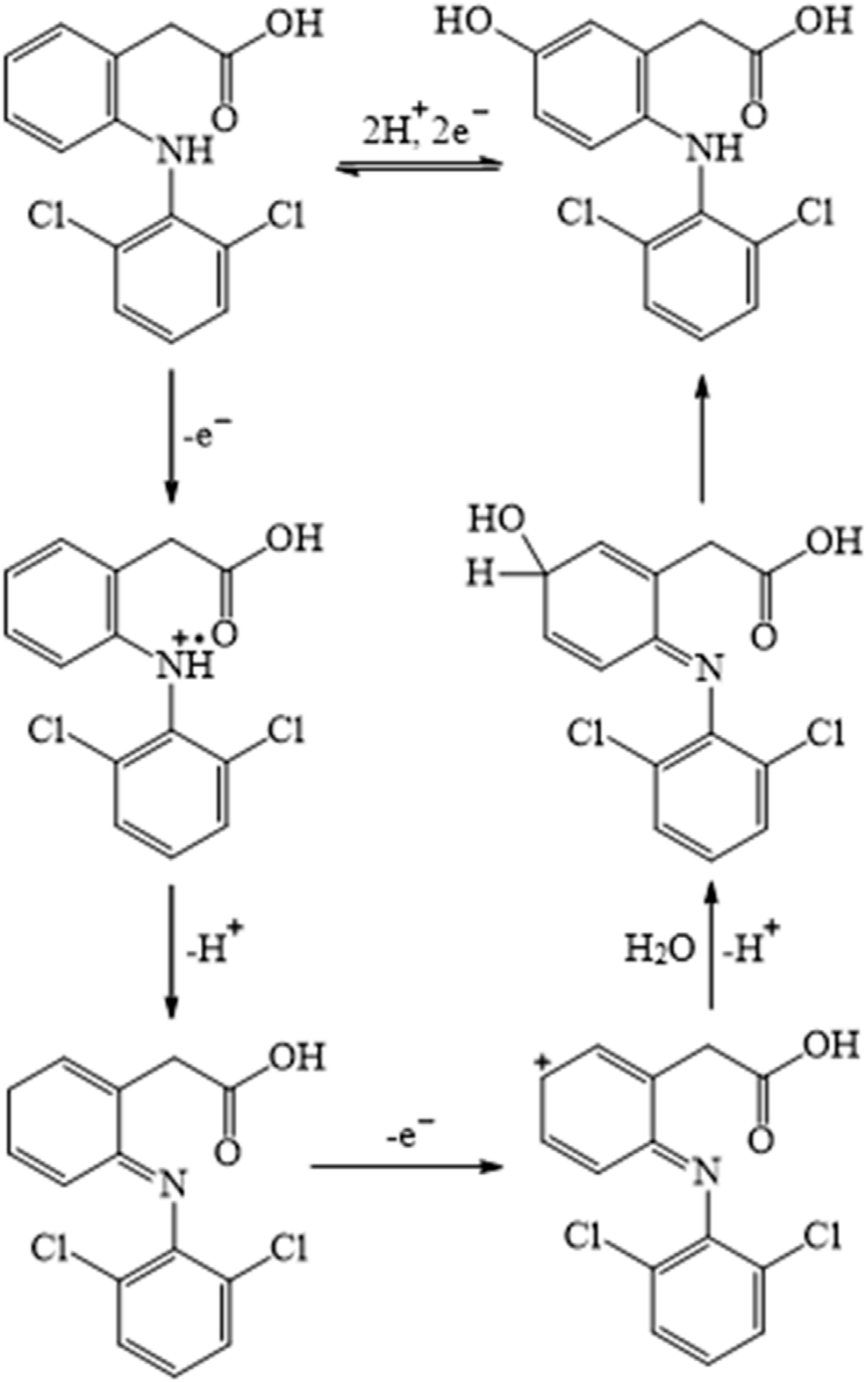
Plausible mechanism of electrooxidation of diclofenac at the modified electrode.

**SCHEME 2:**
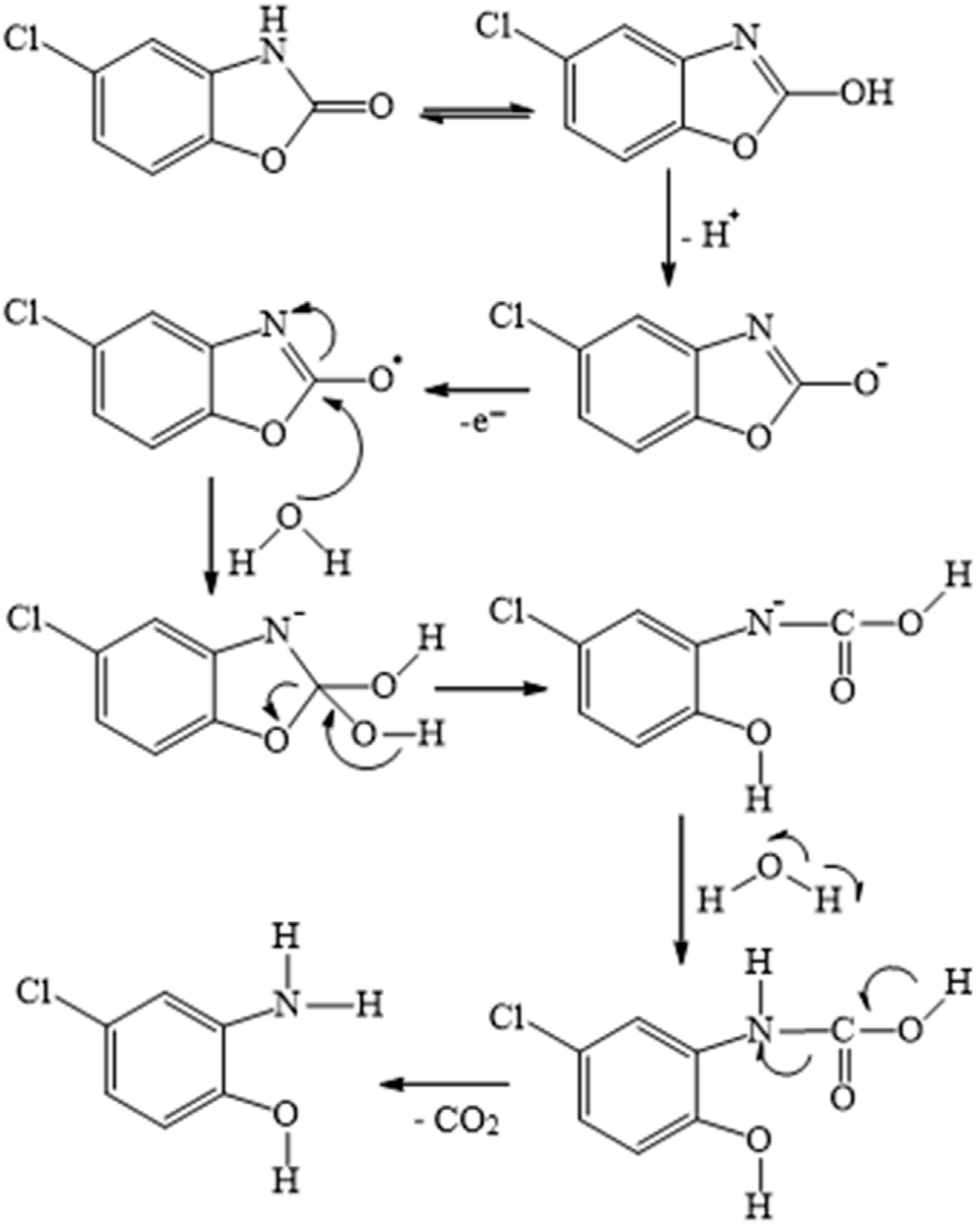
Plausible mechanism of electrooxidation of chlorzoxazone at the modified electrode.

### 3.5 The Scan Rate Effect on Electrochemical Responses of Diclofenac and Chlorzoxazone

**FIGURE 9:**
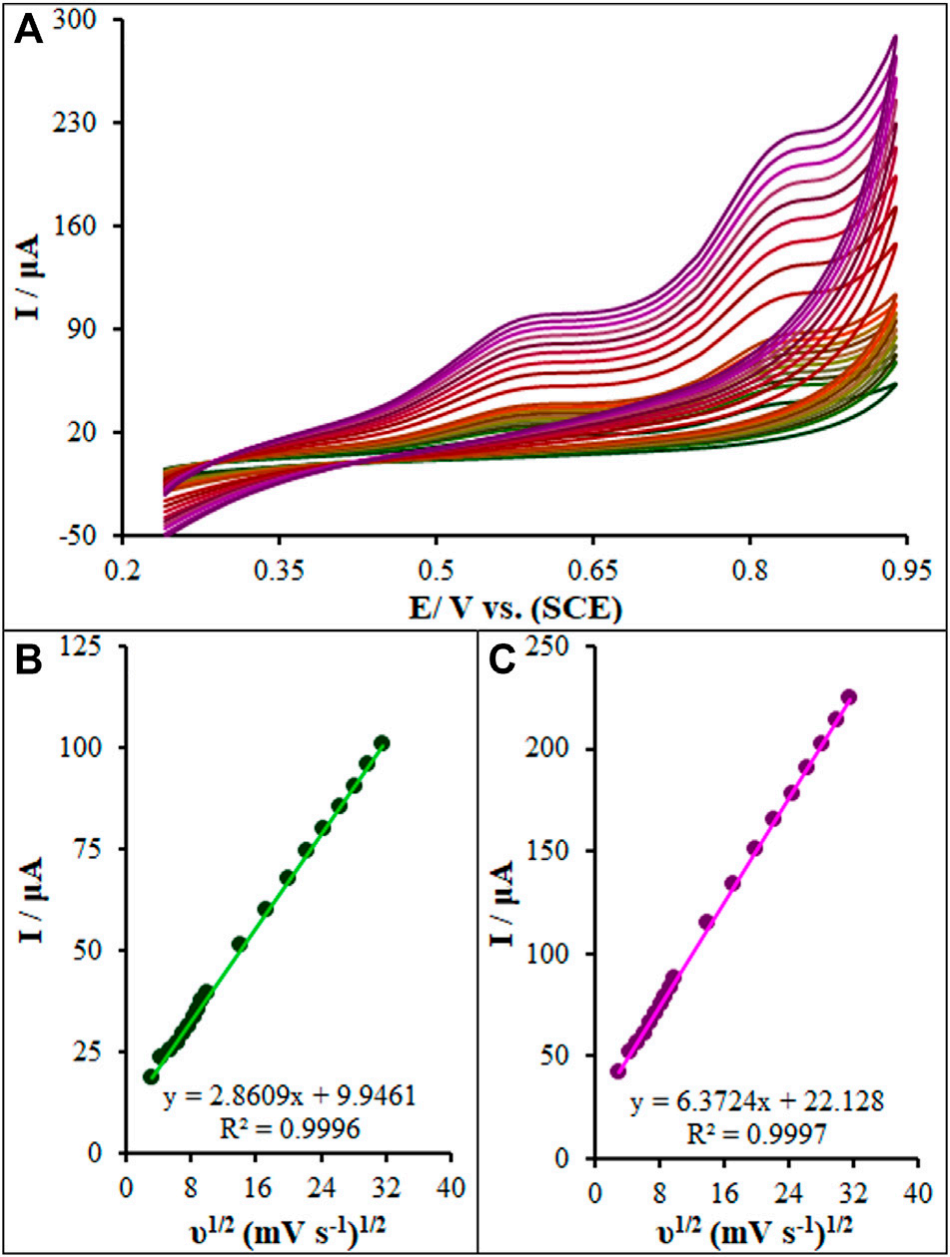
**(A)** CVs of La_2_O_3_@SF-L Cu_2_S/GCE in pH 7.0 in the presence of diclofenac (275.0 µM) and chlorzoxazone (275.0 µM) at various scan rates (from inner to outer curve): 10, 20, 30, 40, 50, 60, 70, 80, 90, 100, 200, 300, 400, 500, 600, 700, 800, 900, and 1000 mV/s. **(B)** The plots of peak currents vs υ^1/2^.

The CV method was employed to evaluate the scan rate effect on the oxidation peak current of diclofenac and chlorzoxazone on the La_2_O_3_@SF-L Cu_2_S/GCE. Figure 9A shows an elevation in the intensity of peak current by raising the scan rate. Based on Figure 9B, the current directly fitted the square root of scan rates (10–1000 mV/s), strongly suggesting diffusion-controlled redox reactions of diclofenac and chlorzoxazone. At last, 50 mV/s was selected as the optimal scan rate to achieve the best performance for peak currents and peak separation.

### 3.6 The Chronoamperometric Measurements

**FIGURE 10:**
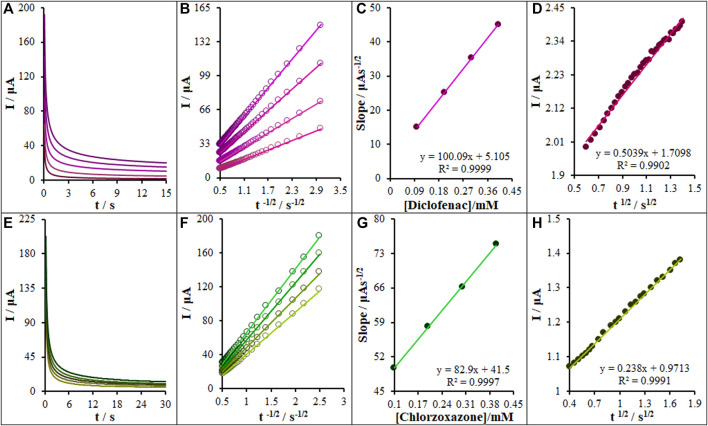
**(A)** Chronoamperograms obtained at La_2_O_3_@SF-L Cu_2_S/GCE in 0.1 M PBS (pH 7.0) for different concentrations of diclofenac (from inner to outer curve): 0.0, 0.1, 0.2, 0.3, and 0.4 mM. **(B)** Plots of I vs t^−1/2^ obtained from chronoamperograms 2–5. **(C)** The plot of the slope of the straight lines against diclofenac concentration. **(D)** The plot of IC/IL vs. t^1/2^ obtained from chronoamperograms one and two. **(E)** Chronoamperograms obtained at the La_2_O_3_@SF-L Cu_2_S/GCE in 0.1 M PBS (pH 7.0) for different concentrations of chlorzoxazone (from inner to outer curve): 0.0, 0.1, 0.2, 0.3, and 0.4 mM. **(F)** Plots of I vs t^−1/2^ obtained from chronoamperograms 2–5. **(G)** The plot of the slope of the straight lines against chlorzoxazone concentration. **(H)** The plot of IC/IL vs. t^1/2^ obtained from chronoamperograms one and two.

The chronoamperometric measurements of diclofenac and chlorzoxazone on the La_2_O_3_@SF-L Cu_2_S/GCE were performed in the working electrode potential at 0.63 and 0.89 V *versus* SCE for different diclofenac and chlorzoxazone concentrations, respectively, in PBS at the pH value of 7.0 (Figures 10A,E). For an electrical agent with a certain diffusion coefficient (D), the Cottrell equation was considered to describe the electrochemical reaction current with a mass transport-limited rate (Bard and Faulkner, 2001).

$$I=nFAD^{1/2}C_{b}\pi^{-1/2}t^{-1/2}$$. (3)

A plot of I *versus* t^−1/2^ is linear under diffusion control (Figures 10B,F); the D value for diclofenac and chlorzoxazone can be calculated on the basis of the linear region slope of Cottrell’s plot (Figures 10C,G). The D_Diclofenac_ and D_Chlorzoxazone_ values were, respectively, 1.16 × 10^−5^ and 3.2 × 10^−5^ cm^2^/s.

Chronoamperometry was recruited to compute the constant of the catalytic rate (*k*) for the reaction of the La_2_O_3_@SF-L Cu_2_S/GCE with diclofenac and chlorzoxazone based on the Galus method (Galus, 1976):

ICIL=γ12[P12erf(γ12)+exp(−γ)γ12], (4)

where *I*_C_ stands for diclofenac and chlorzoxazone catalytic currents on the La_2_O_3_@SF-L Cu_2_S/GCE, *I*_L_ for limiting current without diclofenac and chlorzoxazone, and γ = *kC*_b_ *t* (*C*_b_ for bulk diclofenac and chlorzoxazone concentration, the argument of error function. If the value of γ is greater than 2, the error function will be approximately equal to 1, so the aforementioned equation is decreased to

ICIL=π12γ12=π12(KCbt)12, (5)

where *t* stands for elapsed time (in seconds). Accordingly, the slope from the plot of *I*_C_/*I*_L_ *versus t* ^1/2^ is recruited to compute the catalytic process constant (*k*) for the concentrations of diclofenac and chlorzoxazone. Figures 10E,H show the plots from chronoamperograms in Figures 10A,E. Based on the slopes, the mean values of *k* were 8.1×10^3^ and 1.8 × 10^3^ M^−1^ s^−1^. The *k* value can explain the sharp property of the catalytic peak for diclofenac and chlorzoxazone catalytic oxidation on the La_2_O_3_@SF-L Cu_2_S/GCE surface.

### 3.7 Quantification of Diclofenac and Chlorzoxazone

**FIGURE 11:**
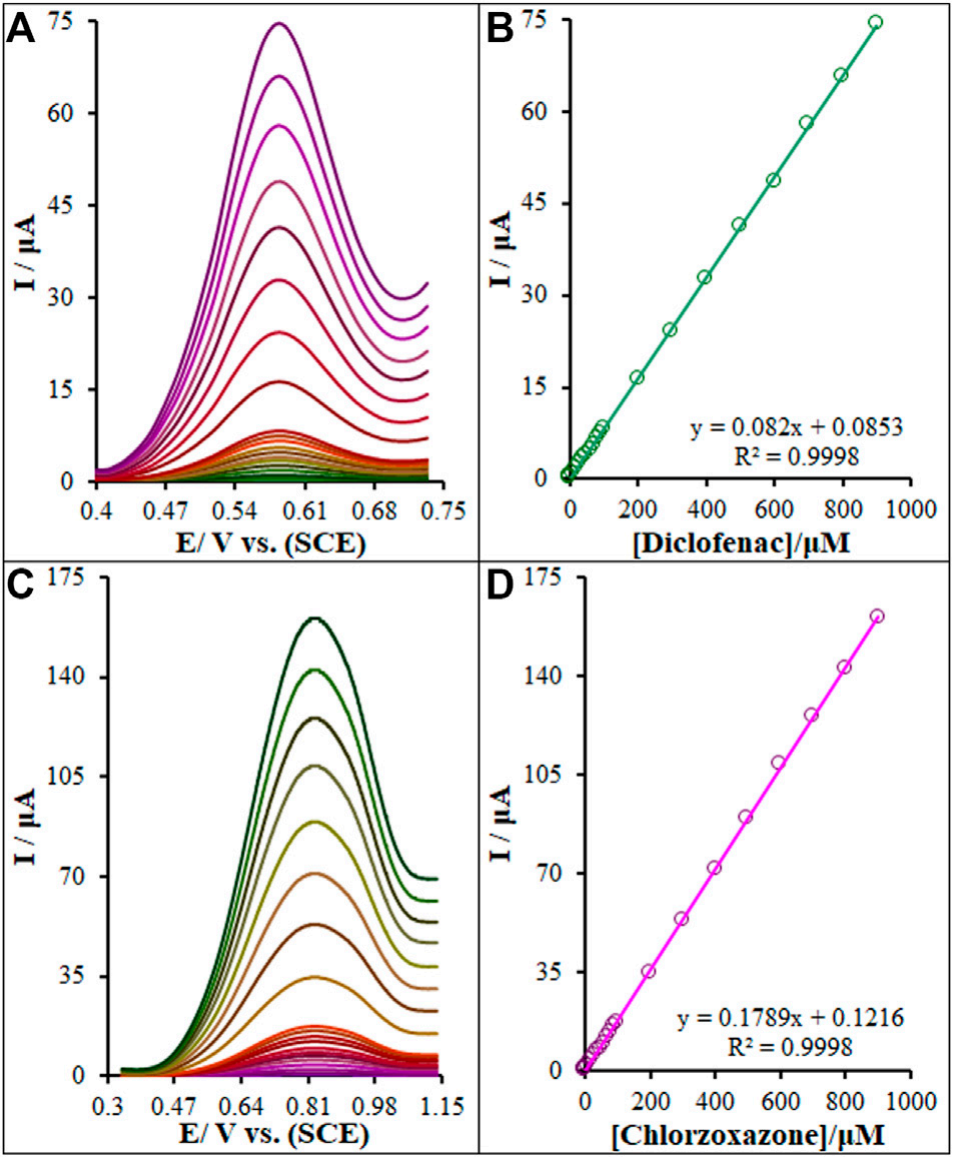
**(A)** and **(C)** DPVs of the La_2_O_3_@SF-L Cu_2_S/GCE in 0.1 M (pH 7.0) containing different concentrations of diclofenac and chlorzoxazone, respectively (from inner to outer curve): 0.01, 0.1, 1.0, 10.0, 20.0, 30.0, 40.0, 50.0, 60.0, 70.0, 80.0, 90.0, 100.0, 200.0, 300.0, 400.0, 500.0, 600.0, 700.0, 800.0, and 900.0 µM. **(B)** and **(D)** Plots of the electrocatalytic peak currents as a function of diclofenac and chlorzoxazone concentrations in the range of 0.01–900.0 µM, respectively.

Diclofenac and chlorzoxazone quantifications were performed using differential pulse voltammetry (DPV) (Figures 11A,C). One clear linear segment with varied slopes can be observed on voltammograms related to the plot of diclofenac and chlorzoxazone concentration *versus* peak current, as seen in Figures 11B,D. Electrocatalytic peak currents from the oxidation of diclofenac and chlorzoxazone on the La_2_O_3_@SF-L Cu_2_S/GCE surface had a linear correlation with different concentrations of diclofenac and chlorzoxazone (0.01–900.0 μM). The limit of detections (LOD, 3S_b_/m) were computed to be 1.7 and 2.3 nM for diclofenac and chlorzoxazone, respectively, where S_b_ stands for the standard deviation of blank and m for the slope of calibration plot. In addition, the limit of quantifications (LOQ, 3S_b_/m) were calculated to be 5.7 and 7.6 nM for diclofenac and chlorzoxazone, respectively.

### 3.8 Simultaneous Detection of Diclofenac and Chlorzoxazone

**FIGURE 12:**
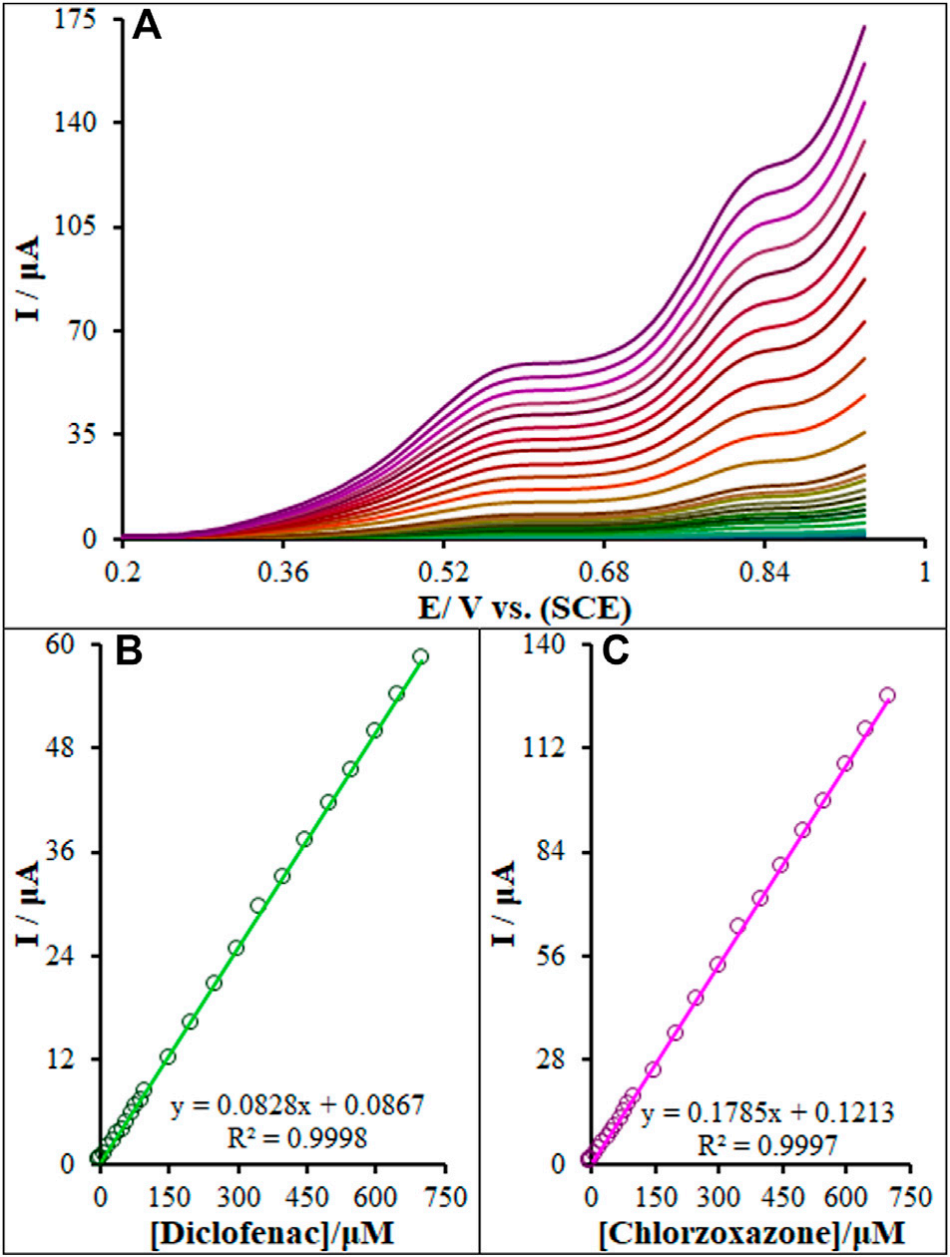
**(A)** DPVs of La_2_O_3_@SF-L Cu_2_S/GCE in 0.1 M (pH 7.0) containing different concentrations of diclofenac and chlorzoxazone (from inner to outer curve): 0.01, 0.1, 1.0, 10.0, 20.0, 30.0, 40.0, 50.0, 60.0, 70.0, 80.0, 90.0, 100.0, 150.0, 200.0, 250.0, 300.0, 350.0, 400.0, 450.0, 500.0, 550.0, 600.0, 650.0, and 700.0 µM. **(B)** and **(C)** Plots of the electrocatalytic peak currents as a function of diclofenac and chlorzoxazone concentrations in the range of 0.01–700.0 µM, respectively.

According to Figure 12A, we found two well-separated peaks and two peak currents increased linearly with the increase of the concentrations of diclofenac and chlorzoxazone. This demonstrates the feasibility of the simultaneous determination of the aforementioned species in the mixture solution applying DPV. The sensitivity of the modified electrode toward the oxidation of diclofenac and chlorzoxazone was found to be 0.082 μA μM^−1^ and 0.1789 μA μM^−1^, respectively, which is very close to the values obtained in the absence of another one’s (0.0828 μA μM^−1^ and 0.1785 μA μM^−1^, see Section 3.7) (Figures 12B,C). These results are indicative that the oxidation processes of these compounds at the La_2_O_3_@SF-L Cu_2_S/GCE are independent, and therefore, simultaneous determination of their mixtures is possible without significant interferences. Five consecutive detections of diclofenac and chlorzoxazone (100.0 μM) showed a relative standard deviation (RSD%) of 1.8 and 2.2, respectively, confirming the effectiveness of the proposed electrode for the simultaneous detection of diclofenac and chlorzoxazone.

### 3.9 Interference Measurements

**FIGURE 13:**
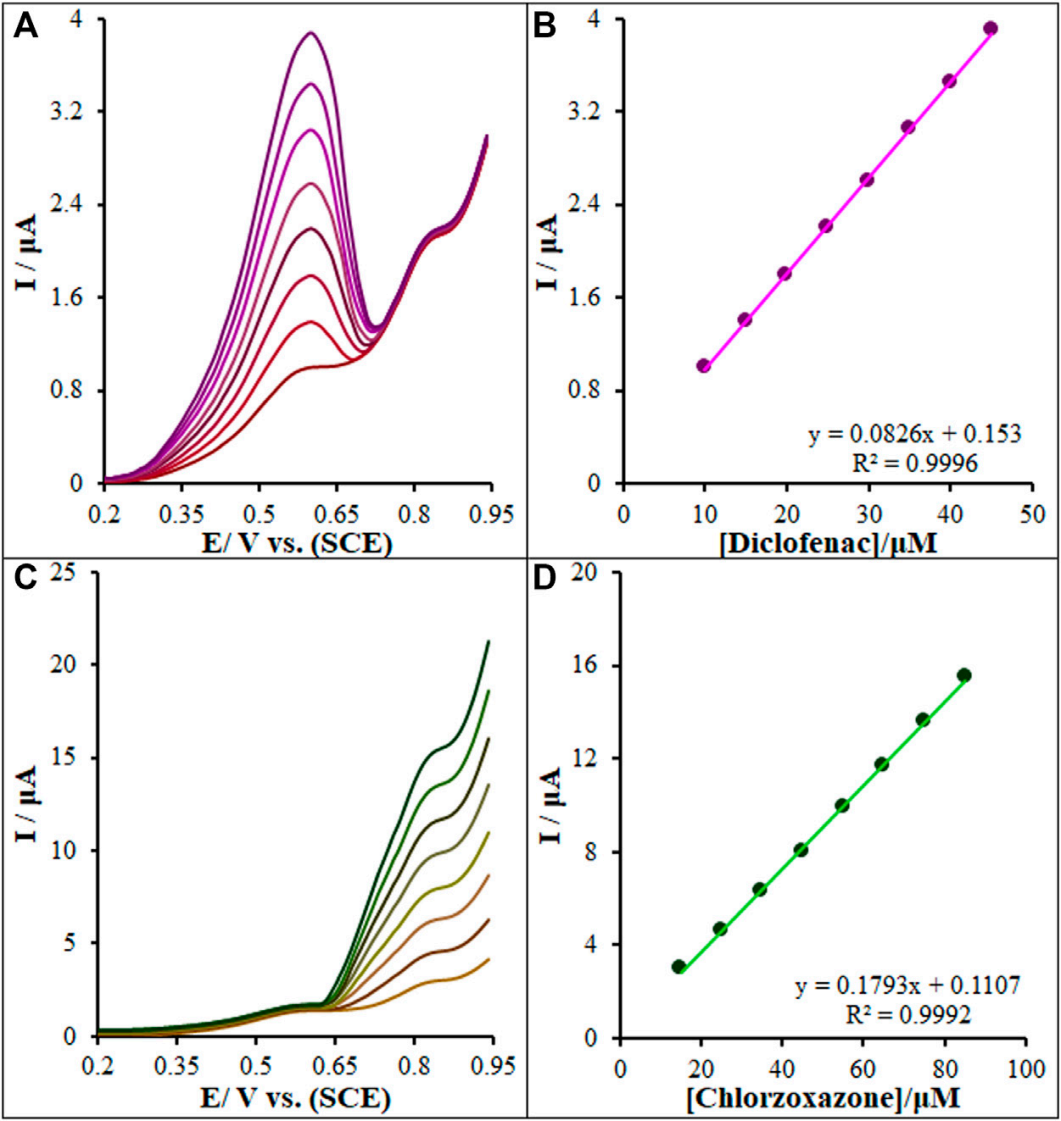
**(A)** DPVs of La_2_O_3_@SF-L Cu_2_S/GCE in 0.1 M (pH 7.0) containing 15.0 µM of chlorzoxazone, and different concentrations of diclofenac (from inner to outer curve): 10.0, 15.0, 20.0, 25.0, 30.0, 35.0, 40.0, and 45.0 µM. **(B)** Analytical curve from diclofenac. **(C)** 15.0 µM of diclofenac, and different concentrations of chlorzoxazone (from inner to outer curve): 15.0, 25.0, 35.0, 45.0, 55.0, 65.0, 75.0, and 85.0 µM. **(D)** Analytical curve from chlorzoxazone.

The coexistence of diclofenac and chlorzoxazone in real samples emphasizes the necessity of examining their interference for the selective determination of one species. In each test, the concentration of a species was variable while keeping constant another one’s concentrations, and the results of which are shown in Figures 13A,C. Figure 13A shows an elevation in the diclofenac oxidation peak current by increasing its concentration, while the chlorzoxazone oxidation peak current was constant. Figure 13B shows that the voltammetric peak of diclofenac oxidation elevated linearly in line by increasing their concentration, but the oxidation peak current of chlorzoxazone was constant. According to the results, peak currents were linearly correlated with diclofenac (or chlorzoxazone) concentrations, while no change occurred in the other compound; this demonstrates the independent implementation of diclofenac and chlorzoxazone oxidation on the La_2_O_3_@SF-L Cu_2_S/GCE.

### 3.10 Analysis of Real Specimens

Diclofenac tablets, human blood serum, and urine samples were analyzed to assess the analytical applicability of the proposed method for simultaneous detection of diclofenac and chlorzoxazone. Thus, the diclofenac and chlorzoxazone levels were measured in these specimens. The samples spiked with a certain level of diclofenac and chlorzoxazone were analyzed to examine the method’s reliability (Table 1). The obtained recovery was between 97.4% and 102.4% for diclofenac and chlorzoxazone, confirming the applicability of the proposed electrode for simultaneous detection of diclofenac and chlorzoxazone.

**TABLE 1:** Application of La_2_O_3_@SF-L Cu_2_S/GCE for concurrent determination of diclofenac and chlorzoxazone in diclofenac tablets, human blood serum, and urine samples. All concentrations are in µM.

Sample	Spiked		Found^a^		Recovery (%)	
	Diclofenac	Chlorzoxazone	Diclofenac	Chlorzoxazone	Diclofenac	Chlorzoxazone
Diclofenac tablets	-	ND^b^	3.8 ± 2.3	-	-	-
	5.0	5.0	9.0 ± 2.2	4.9 ± 1.8	102.3	98.0
	10.0	10.0	9.9 ± 1.9	10.1 ± 3.2	99.0	101.0
Human blood serum	ND^b^	ND^b^	-	-	-	-
	7.5	15.0	7.4 ± 3.1	14.7 ± 2.2	98.6	98.0
	12.5	20.0	12.8 ± 2.1	20.1 ± 2.9	102.4	100.5
Urine	ND^b^	ND^b^	-	-	-	-
	15.5	15.0	15.1 ± 2.7	15.2 ± 1.9	97.4	101.3
	17.5	25.0	17.7 ± 2.8	24.8 ± 1.6	101.1	99.2

a Mean ± standard deviation for n = 5.

b Not detect.

### 3.11 Reproducibility, Repeatability, and Stability

The DPV method was used to study the stability, reproducibility, and repeatability of the electrochemically fabricated sensor under optimized conditions. After six consecutive applications of the La_2_O_3_@SF-L Cu_2_S/GCE to measure the 10.0 µM of diclofenac and chlorzoxazone solution, no distinct change was found in the DPV response. The relative standard deviation (RSD%) of 0.94% confirmed the satisfactory repeatability of the proposed sensor. The DPV method was performed additionally to assess the electrode reproducibility. Six consecutive measurements were carried out to evaluate reproducibility, followed by calculating RSD%. The intra-electrode and interelectrode RSD% was about 1.25% and 2.83%, respectively. The stability of the modified electrochemical sensor was analyzed as well. The electrodes were left in an ambient room for 3 weeks, and there was no significant fluctuation in peak current (2.6%), confirming the appropriate stability of the La_2_O_3_@SF-L Cu_2_S/GCE under optimized conditions. The selectivity of the method was evaluated by testing the 10.0 µM diclofenac and chlorzoxazone solution in exposure to several compounds with potential interferents containing 100-fold sucrose, ascorbic acid, citric acid, dopamine, vitamin B_6_, uric acid, vitamin B_2_, glucose, and starch. According to the results (the signal change of less than 3%), no significant interference was seen between those compounds and diclofenac and chlorzoxazone detection. The proposed method subsequently demonstrated a considerable selectivity for diclofenac and chlorzoxazone detection.

### 3.12 Comparison of Our Method With Others in the Literature

The comparison of analytical efficacy between the as-fabricated electrode and other electrochemical methods was performed individually for each of diclofenac and chlorzoxazone (Tables 2). Based on Table 2, the performance of our proposed electrochemical electrode for sensing diclofenac and chlorzoxazone displayed a comparable linear range, and better detection limit and sensitivity than other methods (Goyal et al., 2010; Abbar and Nandibewoor, 2012a; Abbar and Nandibewoor, 2012b; Chethana et al., 2012; Ihos et al., 2012; Razmi et al., 2013; Honakeri et al., 2020; Zayed and Issa, 2020; Meti et al., 2021; Ngoc Hoa et al., 2021). Accordingly, the as-fabricated sensor is potentially able to determine the trace amounts of studied drugs in various media. Moreover, the electrode used for sensor fabrication is a GCE that has various advantages like cost-effectiveness, facile modification, admirable accessibility, and lower background current when compared with other electrodes such as diamond and carbon paste electrodes. As seen in Table 2, the electrode as-fabricated for electrochemically sensing diclofenac and chlorzoxazone generally showed admirable properties for measurement speed, sensitivity, detection limit, linear range, and sensitivity when compared with other electrochemical methods reported in the literature.

**TABLE 2:** Performance comparison of La_2_O_3_@SF-L Cu_2_S/GCE for the simultaneous determination of diclofenac and chlorzoxazone with other electroanalytical methods.

Electrode	Linear range (µM)	Detection limit	References
Diclofenac			
ZnO@Cu nanoparticles/GCE	0.01–300.0	0.0341 µM	Honakeri et al. (2020)
Plane pyrolytic graphite electrode	0.01–10.0	6.2 nM	Goyal et al. (2010)
Zeolite imidazolate framework-67/graphitic carbon nitride/GCE	0.2–2.2	0.071 µM	Ngoc Hoa et al. (2021)
Tyrosine/carbon paste electrode	10.0–140.0	3.28 µM	Chethana et al. (2012)
Boron doped diamond electrode	0.31–31.1	0.03 µM	Ihos et al. (2012)
Multiwalled carbon nanotube and ionic liquid/carbon ceramic electrode	0.005–20.0	27.0 nM	Razmi et al. (2013)
La_2_O_3_@SF-L Cu_2_S/GCE	0.01–900.0	1.7 nM	This study
Chlorzoxazone			
GCE	0.8–10.0	0.0441 µM	Abbar and Nandibewoor, (2012a)
Gold electrode	5.0–100.0	4.5 µM	Abbar and Nandibewoor, (2012b)
Nanostructured Au/graphene/GCE	0.1–100.0	0.012 µM	Meti et al. (2021)
Carbon paste electrode	0.17–1.68 μg/ml	0.05 μg/ml	Zayed and Issa, (2020)
La_2_O_3_@SF-L Cu_2_S	0.01–900.0	2.3 nM	This study

## 4 Conclusion

The current study aimed to develop a La_2_O_3_@SF-L Cu_2_S composite-modified glassy carbon electrode for simultaneous detection of diclofenac and chlorzoxazone in biological and pharmaceutical specimens. An excellent electrocatalytic potential was obtained for the proposed electrochemical sensor toward the oxidations of diclofenac and chlorzoxazone because of the synergetic activity of La_2_O_3_ and SF-L Cu_2_S on the electrode surface. The applicability of our modified electrode was examined for successful simultaneous detection of diclofenac and chlorzoxazone in drug tablets and urine specimens.

## Data Availability

The original contributions presented in the study are included in the article/Supplementary Material; further inquiries can be directed to the corresponding authors.
